# Identify the origin of *de novo* variants in TSC patients by ddPCR

**DOI:** 10.1186/s42494-025-00227-1

**Published:** 2025-08-01

**Authors:** Kun Ni, Xiaolong Yu, Jiehui Ma, Dan Sun

**Affiliations:** 1 Department of Neurology, Wuhan Children’s Hospital, Tongji Medical College, Huazhong University of Science & Technology, Wuhan, 430014 China; 2 Department of Emergency and Critical Care Center, Wuhan Children’s Hospital, Tongji Medical College, Huazhong University of Science & Technology, Wuhan, 430014 China

**Keywords:** Whole-exome sequencing, *TSC1*/*TSC2* panel, DdPCR, Mosaic

## Abstract

**Background:**

Tuberous sclerosis complex (TSC), an inherited neurocutaneous disorder, is caused by variants in the *TSC1* or *TSC2* genes. The mosaic variants of TSC1 and *TSC2* are scarcely detectable using the conventional next-generation sequencing (NGS). Therefore, this study aims to explore the detection and distribution of mosaic variants within affected families.

**Methods:**

Through whole-exome sequencing (WES) or the *TSC1*/*TSC2* panel to detect the variants of the *TSC1* and *TSC2* genes, the reaction system of droplet digital PCR (ddPCR) was designed to detect the mosaicism of these variants in affected families.

**Results:**

Genetic testing was carried out on 29 TSC patients via WES or the *TSC1*/*TSC2* panel. The results showed that 27 patients had positive results in the *TSC* gene variant tests. Fourteen cases were confirmed as de novo variants, and the asymptomatic fathers or mothers of 4 patients were identified as somatic mosaics by ddPCR, with mosaic proportions of 0.8%, 24.18%, 8.02%, and 0.33% respectively.

**Conclusions:**

The ddPCR holds the potential to improve diagnostic accuracy, genetic risk assessment, and clinical diagnosis rates. Consequently, it could potentially be adopted as one of the modalities for prompt clinical diagnosis.

**Supplementary Information:**

The online version contains supplementary material available at 10.1186/s42494-025-00227-1.

## Background

Tuberous sclerosis complex (TSC) is an autosomal dominant neurocutaneous syndrome characterized by involvement of multiple organ systems [1]. The two principal disease-causing genes in TSC are *TSC1* and *TSC2*, which encode the hamartin protein and tuberin protein, respectively. Pathogenic variants lead to structural changes in the proteins that prevent the hamartin protein and tuberin proteins from heterodimerization. These variant-induced functional changes, manifested as disrupted heterodimerization, ultimately give rise to the clinical phenotypes associated with TSC [2]. The main structural disorder in the nervous system are cortical dysplasia, subventricular nodules, and subventricular giant cell astrocytoma. Consequently, the clinical manifestations of TSC are complex and diverse, often presenting with the classic clinical triad of epilepsy, intellectual disability and facial sebaceous adenoma. The earlier the onset of seizure, the greater the risk of comorbid severe neuropsychiatric deficits [3]. Early detection and control of seizures in children with TSC are crucial for enhancing their prognosis. This is an important factor in improving their quality of life and intellectual development. Children with *TSC2* variants tend to develop epilepsy significantly earlier compared to those with *TSC1* variants. Moreover, they are more likely to experience infantile spasms and have poorer epilepsy control and neurodevelopmental outcomes than their counterparts with *TSC1* variants [4].

*TSC1* maps to chromosome 9q34 and comprises 23 exons, encoding an 8.6-kb transcript. *TSC2* localizes to chromosome 16p13 and contains 42 exons, giving rise to a 5.5-kb transcript. In conjunction with the TBC1D7 protein, these genes form a heterotrimeric complex that modulates the mTOR signaling pathway. *TSC1*/*TSC2* panel sequencing, Whole-exome sequencing (WES) and multiplex ligation-dependent probe amplification (MLPA) are capable of detecting heterozygous single-nucleotide variants, indels, and duplications in all exons and intronic-regions adjacent to exons. This has long been proven widely effective in numerous studies [5–8]. Long-range PCR and quantitative PCR have also been applied in the detection [9, 10]. However, 10–15% of clinically diagnosed TSC patients remain genetically unresolved using these approaches, as no pathogenic variants are identified. Notably, the detection rate of *TSC1*/*TSC2* variants can be improved by increasing the depth of sequencing [11]. Variants in deep intronic regions may require detection methods such as long-range PCR and whole-genome sequencing (WGS). Treichel et al. reported that 58% of all variants identified mosaic variants, and intronic variants accounted for 40% [12]. These mosaic variants involved somatic variants in DNA extracted from angiofibroma biopsies and low-level mosaic variants in DNA isolated from blood or saliva. However, the clinical relevance of these mosaic variants, particularly their association with phenotypic expression, requires further investigation.

Droplet digital PCR (ddPCR) represents a new-generation quantitative polymerase chain reaction, which significantly diverges from traditional PCR technology. Prior to sample amplification, the sample undergoes droplet formation. Specifically, the reaction system is partitioned into thousands of nanoliter-sized droplets. Each droplet functions as an independent reaction unit and may contain zero or multiple copies of the target DNA. Ultimately, in accordance with the principle of Poisson distribution, by integrating the quantity and proportion of fluorescent droplets, the initial concentration of the target to be detected can be derived [13]. This method had been widely applied in the research related to viruses [14, 15] and tumors [16], and it is also gradually being used in the research of mosaic variants [17, 18]. Interestingly, Wang et al. applied ddPCR to the clinical research of TSC for the first time in a previous study [11].

This report is designed to investigate the variation status of *TSC* genes within the patient's family via NGS, and to examine the mosaicism of these variants among the family members through ddPCR. The intention is to preclude the oversight of clinical manifestations and excessive dependence on NGS results. Utilizing high-depth gene panels or ddPCR assays targeting known variants can assist clinicians in the early detection of genetic aberrations associated with *TSC* mosaicism. This holds substantial implications for the reassessment of clinical symptoms in the tested individuals and provides valuable guidance for prospective reproductive decision-making.

## Methods

### Participants

We recruited 29 TSC patients who presented at our hospital between January 2018 and May 2019. All patients were diagnosed according to the diagnostic criteria of the TSC Consensus Conference [19]. The inclusion criteria consisted of a definite clinical diagnosis of TSC and the absence of prior genetic testing before recruitment [8]. Excluded patients who had been previously diagnosed with TSC via genetic testing and those who were unwilling to cooperate with subsequent genetic testing.

### DNA extraction

The genomic DNA was extracted from the peripheral blood samples of both the proband and parents using the QIAamp® Blood Mini Kit (Qiagen, Germany), followed by concentration and purity measurement with a NanoDrop™ 2000 spectrophotometer (Thermo Fisher Scientific, USA).

### Whole-exome sequencing

Genomic DNA fragmentation into 150–300 bp segments was achieved via a Covaris M220 ultrasonicator (Covaris, USA). The sheared DNA underwent end-repair and adenylation, followed by adapter ligation and amplification steps. Targeted enrichment of the amplified library was performed using the xGen® Exome Research Panel v1.0 kit (IDT, USA). Paired-end 150 bp sequencing (PE150) was conducted on the MGISEQ-2000 platform (MGI, China). Bioinformatics analyses were executed using an in-house pipeline incorporating publicly available software tools. Specifically, raw reads were first preprocessed to discard low-quality sequences and adapter contaminants via fastp 0.20.0 [20]. Read alignment was then performed using the Burrows-Wheeler Aligner tool 0.7.17 with the BWA-MEM algorithm (default parameters), referencing the human G1 Kv37 genome assembly [21, 22]. Resulting bam files were sorted using SAMtools 1.9 [23], and duplicate reads were removed via Picard 2.19.0. Germline variant calling was performed using the GATK Haplotyper algorithm within Sentieon’s genomics package v.201911 [24].

### High-depth TSC panel sequencing

The amplified library was subjected to targeted capture using the *TSC1*/*TSC2* panel, and the sequencing workflow was identical to that of WES. The coverage ratio of the *TSC1*/*TSC2* gene regions was higher than 99%, and the average sequencing depth exceeded 3000 ×. The original sequencing data were first subjected to quality control and cleaning by fastp v0.20.0 to generate high-quality data [20]. Subsequently, Sentieon v201911 was used to align the cleaned sequences to the reference genome hg19, generating BAM alignment files [25]. On this basis, Sentieon and VarDict v1.8.3 [26] were employed to conduct single nucleotide variation (SNV) detection in the regions of *TSC1* (chr9:135766735–135820094) and *TSC2* (chr16:2097472–2138713), and Ensembl-VEP v100.4 was used to perform functional annotation on the mutation results [27]. Meanwhile, CNVkit v0.9.7 [28] was utilized to conduct copy number variation (CNV) detection in the *TSC*1 and *TSC2* regions, and AnnotSV v2.0 [29] was adopted to further annotate the CNV results.

### Droplet digital PCR (ddPCR)

In this study, the QX200™ Droplet Digital™ PCR System (BioRad, USA) was operated following the manufacturer’s protocols. Genomic DNA was extracted from blood samples prior to analysis. The ddPCR reaction mixtures (20 μL) were prepared by combining 10 μL ddPCR Supermix, 10 µM of each primer, 10 µM of the probe, and 5.4 μL of sample. The thermocycling conditions consisted of an initial denaturation step at 95 °C for 10 min, followed by 40 cycles of denaturation at 94 °C for 30 s, annealing at 60 °C for 60 s, and a final incubation at 98 °C for 10 min, with samples stored at 4 °C thereafter. The droplets of each reaction were sequentially analyzed using a QX200™ Droplet Reader, which enabled direct data upload to a computer for final statistical processing.

## Results

A total of 29 patients, along with their parents, were tested by WES or the *TSC1*/*TSC2* panel. Twenty-seven patients had positive genetic test results. Among them, pathogenic variants were found in the *TSC1* gene in 9 patients (33.3%), and in the *TSC2* gene in the remaining 18 patients (66.7%). Based on the results of Next-Generation Sequencing, 23 patients had presumed de novo variants, and no identical variants were found in their parents. The remaining four patients (P24–P27) had paternal or maternal variants. In this study, only one patient had a copy number variation: Patient 2 had a deletion of exons 1–14 in the *TSC1* gene. The NGS data suggested that the patient's variant was mosaic, with a proportion of approximately 55% (Supplementary Fig. 1). This result was obtained from a high-depth gene panel. Her clinical manifestations were not different from other TSC patients. The remaining variants included 5 missense, 8 intronic, 2 nonsense, and 11 microdeletion/microduplication variants (Table 1).

**Table 1:** Overview of the identified variants of TSC1/TSC2

Patients (Gender)	Gene	Chromosome position (GRCh37/hg19)	cDNA change (Amino acid change)	Inheritance
P1 (M)	*TSC2*	chr16:2126143	c.2714G>A(p.Arg905Gln)	*De novo*
P2 (F)	*TSC2*	chr16:2097425–2113274	Del Exon1-14	Unconfirmed
P3 (M)	*TSC2*	chr16:2137903	c.5029del(p.Asp1677ThrfsTer149)	*De novo*
P4 (M)	*TSC1*	chr9:135786880	c.989dup(p.Ser331GlufsTer10)	*De novo*
P5 (F)	*TSC1*	chr9:135787689–135787690	c.892_893del(p.Ala298Ter)	*De novo*
P6 (M)	*TSC1*	chr9:135781190–135781196	c.1769_1775del(p.Pro590ArgfsTer37)	*De novo*
P7 (F)	*TSC1*	chr9:135781467	c.1498C>T(p.Arg500Ter)	*De novo*
P8 (M)	*TSC2*	chr16:2112497	c.1258-1G>A	Paternal mosaicism
P9 (F)	*TSC1*	chr9:135796749	c.737+1G>T	Maternal mosaicism
P10 (M)	*TSC2*	chr16:2136801	c.4918C>T(p.His1640Tyr)	*De novo*
P11 (M)	*TSC2*	chr16:2126513–2126514	c.2764_2765delTT(p.leu922valfsTer3)	*De novo*
P12 (M)	*TSC2*	chr16:2105525	c.599+5G>A	*De novo*
P13 (M)	*TSC2*	chr16:2130366	c.3598C>T(p.Arg1200Trp)	*De novo*
P14 (M)	*TSC2*	chr16:2103381–2103382	c.264_265del(p.Leu89AlafsTer36)	*De novo*
P15 (F)	*TSC2*	chr16:2138294	c.5227C>T(p.Arg1743Trp)	Paternal mosaicism
P16 (M)	*TSC2*	chr16:2136808	c.4925G>A(p.Gly1642Asp)	*De novo*
P17 (F)	*TSC2*	chr16:2121554–2121563	c.1886_1895del(p.Leu629ProfsTer66)	Paternal mosaicism
P18 (M)	*TSC2*	chr16:2110656	c.976-15G>A	*De novo*
P19 (M)	*TSC1*	chr9:135779843	c.1998-2A>G	*De novo*
P20 (M)	*TSC2*	chr16:2129410	c.3265C>T(p.Gln1089Ter)	Unconfirmed
P21 (M)	*TSC2*	chr16:2126529	c.2780dupC(p.Glu929ArgfsTer11)	Unconfirmed
P22 (F)	*TSC2*	chr16:2136792–2136794	c.4912_4914del(p.Lys1638del)	Unconfirmed
P23 (M)	*TSC1*	chr9:135781074–135781077	c.1888_1891del(p.Lys630GlnfsTer22)	Unconfirmed
P24 (M)	*TSC1*	chr9:135780963–135780966	c.1997+2_1997+5del	Maternal
P25 (F)	*TSC2*	chr16:2098755–2098756	c.138+2_138+3del	Maternal
P26 (M)	*TSC1*	chr9:135772951	c.2672dup(p.Asn891LysfsTer13)	Paternal
P27 (F)	*TSC2*	chr16:2104295	c.337-2A>C	Paternal

Unconfirmed:The parental mosaicism was not determined by ddPCR

**Fig. 1:**
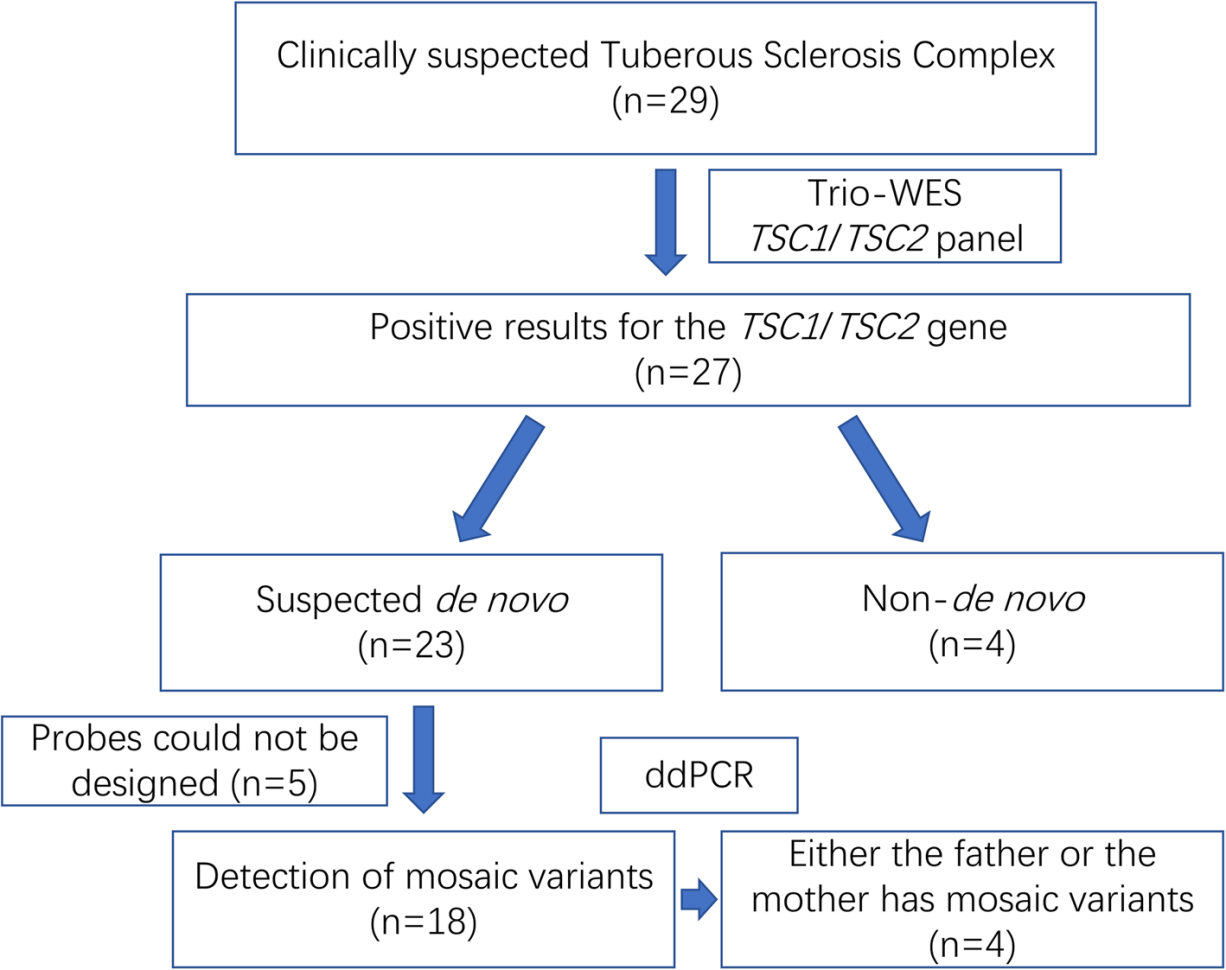
Research flow chart

Subsequently, we intended to conduct ddPCR on 23 patients with presumed de novo variants. Five patients were excluded due to the inability to design probe caused by non-specificity of the target regions. Ultimately, the test results of 18 patients were obtained. Fourteen patients were confirmed to have de novo variants, with no variant signals detected in their parents. Meanwhile, the fathers or mothers of four patients were found to exhibited mosaicism (Fig. 1).

**Fig. 2:**
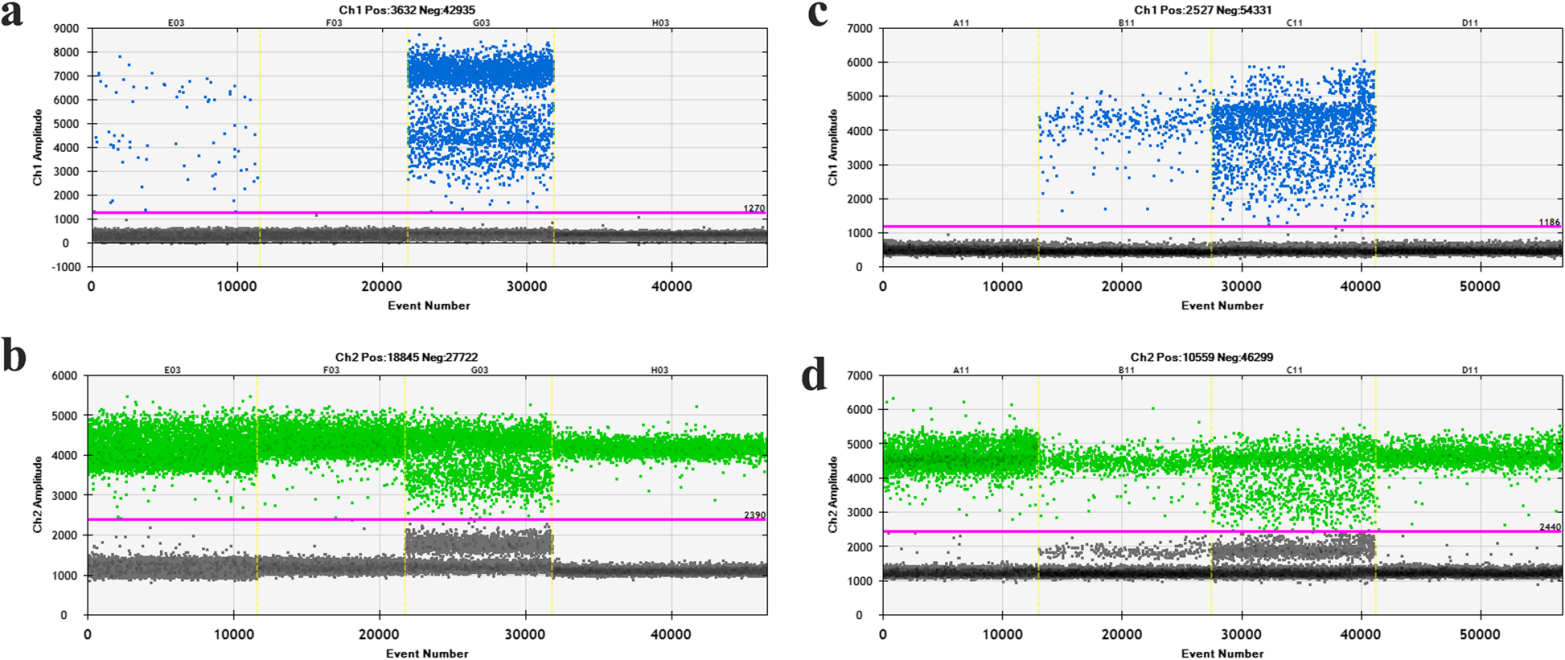
The results of droplet digital PCR (ddPCR) detection for the families of Patient 8 and Patient 9. **a**, **b**, Detection results of mutant and wild-type probes for the families of Patient 8 (E03: Father; F03: Mother; G03: Patient 8; H03: Control). **c**, **d**, Detection results of mutant and wild-type probes for the families of Patient 9 (A11: Father; B11: Mother; C11: Patient 8; D11: Control)

The father of Patient 8 was found to have a mosaic variant of *TSC2*: c.1258-1G>A, with a mosaic proportion of 0.8%. A mosaic mutation with a proportion of 24.18% was identified in the mother of Patient 9 (Fig. 2). Moreover, mosaic variants with proportions of 8.02% and 0.33% were respectively detected in the fathers of Patient 15 and Patient 17 (Supplementary Fig. 2). These findings imply that the variants of these four patients might be inherited from their parents, and relevant tests on germline cells are still required. Interestingly, all of their parents manifested as phenotypically normal, regardless of the level of the mosaic variant proportion.

## Discussion

TSC is a rare autosomal dominant genetic disorder that affects multiple organ systems. It can lead to tumors in important organs such as the brain, eyes, heart, kidneys, liver, and skin. The majority of TSC cases are caused by pathogenic variants in the *TSC1* and *TSC2* genes, with approximately 26% attributed to *TSC1* variants and 69% to *TSC2* variants according to previous literature [30]. In our study, *TSC1* variants accounted for 33.3% of cases while *TSC2* variants accounted for 66.7% (Table 1). These identified gene variants were assessed as pathogenic or likely pathogenic according to the ACMG guidelines. Although clinical criteria support TSC diagnosis, two patients still yielded negative results in WES and TSC panel detection. No definite pathogenic gene variations were found in their families. However, we believe that genetic testing plays an irreplaceable role in assessing the risk of recurrence in affected family members and their offspring. Previous studies had shown that some TSC gene variants were the result of mosaicism. In our study, a mosaic CNV deletion variant in *TSC1* was detected through high-depth gene panel in one pantient presenting focal seizures with core TSC features. It is worth noting that no abnormal variants were found in this patient during WES. We speculate that this might be due to the difficulty in detecting mosaics and exon deletions when using WES. Therefore, for patients with a high clinical suspicion of TSC, it may be necessary to consider supplementary high-depth gene panel testing. In addition, we do not exclude the possibility that certain TSC cases are caused by pathogenic variants in *TSC1*/*TSC2* within the affected tissues. This may require deep sequencing of the lesioned tissues for identification [31].

Only four patients were proven to harbor harmful variants of paternal/maternal origin via WES and TSC panel analyses. We utilized ddPCR to verify all family members of the patients who were positive for *TSC1*/*TSC2*. Of 23 patients, 18 obtained additional de novo verification through ddPCR, and finally, we identified four parental carriers with mosaic variants. However, the parents carrying these mosaic variants, exhibited no TSC-related phenotypes. We speculate that low-level mosaicism (below systemic detection thresholds) may account for this observation. For couples in such situations, their offspring still face a high risk of developing TSC. Therefore, for these patients, we recommend proactive prenatal diagnosis and/or preimplantation genetic testing prior to conception. This will greatly reduce the risk of TSC in future generations, as the presence of *TSC1*/*TSC2* chimeric pathogenic variants in the parental germ cells of the couple can be inferred from their affected progeny [32]. Unfortunately, we did not obtain the parental gonadal cells for direct testing. When they seek future pregnancies, prenatal screening and genetic diagnosis of the embryo are essential.

There are various *TSC* gene diagnostic techniques, including sequencing analyses (e.g., WES and Sanger sequencing), deletion/duplication detection in target regions via multiplex ligation-dependent probe amplification (MLPA) and long-range PCR, as well as ddPCR [10]. As a third-generation technology, ddPCR allows for absolute quantification of nucleic acid molecules [33]. It overcomes the limitations of the second-generation PCR system in accurately determining gene copy numbers, qualitative and quantitative analysis of trace mutations, and low precision. Its enhanced precision and digital partitioning make it suitable for the precise detection of nucleic acid changes in limited samples, thus providing reliable data, particularly in the field of clinical research [14].

However, it is worth noting that limitations of ddPCR should be recognized. It relies on the prior confirmation of variant sites because primers need to be designed for the target regions for the PCR reaction. For completely unknown variants, ddPCR cannot be used for identification. Therefore, it is often necessary obtain gene variant information through WES or gene panel before ddPCR applications. In two families where no potentially harmful variants were detected by WES or the TSC panel, despite the fact that their children were clinically diagnosed with TSC, we were unable to identify possible low-proportion chimeric variants through ddPCR.

In this study, ddPCR was applied as a primary diagnostic modality for TSC patients, demonstrating concordant findings with WES and TSC panel sequencing. For families with negative results detected by WES and TSC panel results, ddPCR successfully identified some abnormal mosaic pathogenic variants in *TSC1*/*TSC2* of the parents. The integrated application of WES with ddPCR improveed the diagnostic rate of *TSC* gene variants, provided a new direction for rapid diagnosis in blood, and offers genetic guidance suggestions for potential fertility needs. Crucially, these findings also rely on the ddPCR detection of gonadal mosaicism in variant carriers. Because some *TSC* variants reside in intronic regions excluded from WES coverage [34], resulting in the inability to establish a definite genetic diagnosis for some clinically diagnosed TSC patients, the cases included in this study were all positive in WES. One case initially yielding negative results revealed partial exon mosaicism upon optimized sequencing depth adjustment. The data did not include pathogenic variants in intronic regions. Further research is required to explore whether ddPCR can increase the diagnostic rate of family members for intronic variants.

In addition, due to the rarity of TSC, the number of cases in this study is limited. More multicenter studies with larger sample sizes would be beneficial in improving the genetic diagnosis and understanding the inheritance patterns of TSC. The relationship between mosaic proportions and clinical phenotypes in parents with chimeras also requires further investigation.

## Conclusions

In summary, regarding the genetic diagnosis of TSC, the implementation of ddPCR can enhance the clinical diagnostic rate and refine genetic risk assessment. This approach provides supplementary genetic diagnostic information for patients and their parents, thereby strengthening the overall diagnostic framework for TSC.

## Supplementary Information

Supplementary Material 1. Supplementary Figure 1. Patient 2 had a mosaic copy number deletion, and no abnormalities were detected in either of the parents

Supplementary Material 2. Supplementary Figure 2. The results of droplet digital PCR (ddPCR) detection for the families of Patient 15 and Patient 17. a, b, Detection results of mutant and wild-type probes for the families of Patient 15 (A01: Mother; B01: Father; C01: Patient 15; D01: Control). c, d, Detection results of mutant and wild-type probes for the families of Patient 17 (E08: Mother; F08: Father; G08: Patient 17; H08: Control)

## Data Availability

Due to the anonymity of the participants/patients, the dataset of this article is not publicly available. Requests to access the dataset should be directed to the corresponding author.

## Abbreviations

CNV: Copy number variation
DDPCR: Droplet digital PCR
MLPA: Multiplex ligation-dependent probe amplification
NGS: Next-generation sequencing
SNV: Single nucleotide variation
TSC: Tuberous sclerosis complex
WES: Whole-exome sequencing
WGS: Whole-genome sequencing
