# Supplementary figures and images for: Identify the origin of de novo variants in TSC patients by ddPCR
Source: Acta Epileptol. 2025 Aug 1;7:37. doi: 10.1186/s42494-025-00227-1 (PMC12315287; doi:10.1186/s42494-025-00227-1)

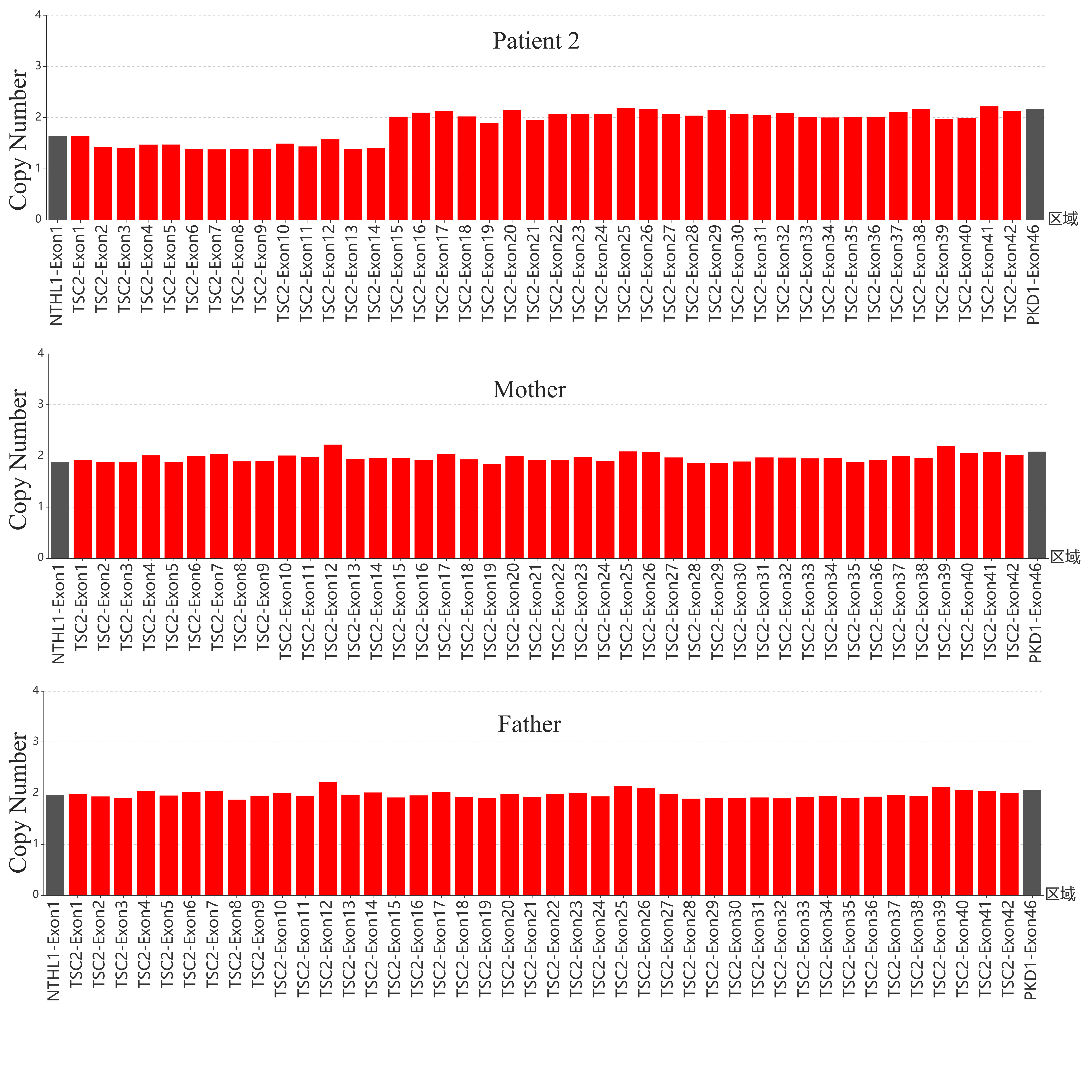

Supplement: Supplementary file 1 — Supplementary Material 1. Supplementary Figure 1. Patient 2 had a mosaic copy number deletion, and no abnormalities were detected in either of the parents [file 42494_2025_227_MOESM1_ESM.tif]

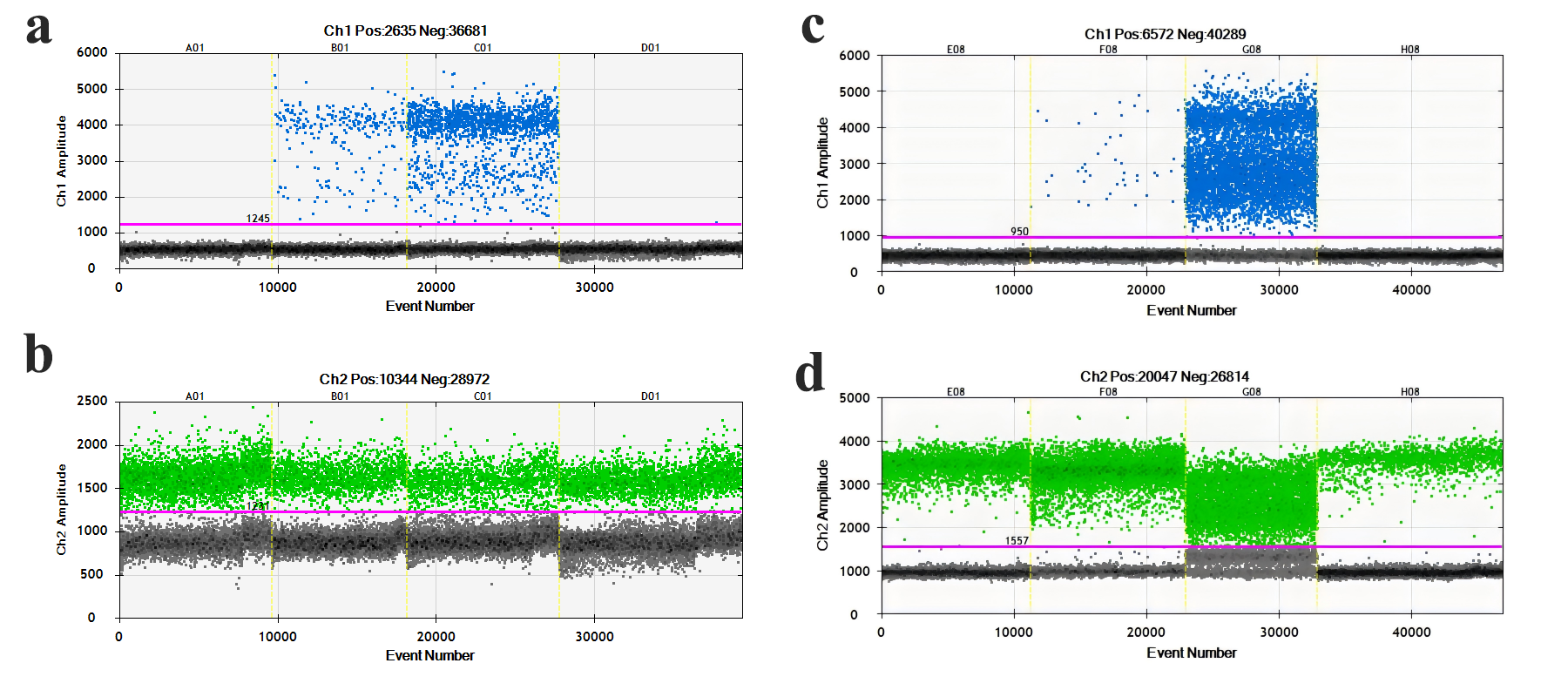

Supplement: Supplementary file 2 — Supplementary Material 2. Supplementary Figure 2. The results of droplet digital PCR (ddPCR) detection for the families of Patient 15 and Patient 17. a, b, Detection results of mutant and wild-type probes for the families of Patient 15 (A01: Mother; B01: Father; C01: Patient 15; D01: Control). c, d, Detection results of mutant and wild-type probes for the families of Patient 17 (E08: Mother; F08: Father; G08: Patient 17; H08: Control) [file 42494_2025_227_MOESM2_ESM.tif]
